# Rare Presentation of Cardiotoxicity Related to 5-Fluorouracil

**DOI:** 10.1155/2020/4151474

**Published:** 2020-07-20

**Authors:** Mariam Charkviani, Natia Murvelashvili, Francisco Barrera, Alisha Sharma, Randa Sharag Eldin, Nur Un Nisa Nabil

**Affiliations:** Amita Saint Francis Hospital, Evanston, IL, USA

## Abstract

5-Fluorouracil (5-FU) is a chemotherapeutic agent frequently used for the treatment of solid tumors. In a few cases, 5-FU can be associated with coronary vasospasm, cardiac ischemia, or life-threatening arrhythmias. Recognition of 5-FU cardiotoxicity is clinically important as after the rapid sensation of therapy, cardiotoxicity can be completely reversible, and on the other hand, readministration may lead to serious damage of the heart and even death. A 70-year-old male came to the emergency department (ED) with chest pain which started while receiving an infusion of 5-FU. The patient did not have a personal history or risk factors of coronary artery disease and his electrocardiogram (ECG) before starting chemotherapy was completely normal. In the ED, his ECG had ischemic changes, troponin was elevated, and echocardiogram showed anterior wall hypokinesis. However, emergent coronary angiogram did not reveal any acute coronary occlusion. 5-FU-induced cardiotoxicity was suspected; the patient was admitted to a progressive care unit for close monitoring and infusion of calcium channel blockers was initiated. The patient's symptoms and ECG findings gradually resolved, and two days later on discharge, patient was chest pain free and ECG was normal. This case supports the vasospastic hypothesis of 5-FU cardiac toxicity, describes its clinical course, and emphasizes the importance of better awareness and early recognition of the rare side effect as it may allow physicians to reduce the risk of life-threatening complications.

## 1. Background

5-Fluorouracil (5-FU) is a chemotherapeutic agent that is frequently used in the treatment of solid tumors. Despite proven therapeutic efficiency, 5-FU can have some undesired side effects, and, in a few cases, it was found to be associated with cardiotoxicity [1–3]. The incidence is rare and according to different sources, it varies from 1 to 19% [4, 5]. The most common clinical manifestation is angina but asymptomatic electrocardiogram (ECG) changes, arrhythmias, myocardial infarction (MI), heart failure, and cardiac arrest have been also reported [3]. We will present the case of a 70-year-old male, who developed coronary vasospasm after starting chemotherapy with 5-FU for newly diagnosed colorectal cancer.

## 2. Case Report

**Figure 1:**
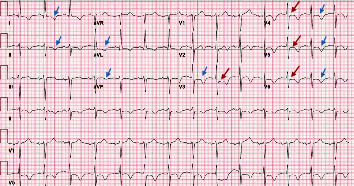
12-lead electrocardiogram (ECG) obtained at the time of arrival of the patient to the ED. ECG shows diffuse *T* wave inversions (blue arrows) and ST depressions in V3, V4, V5, and V6 (red arrows).

**Figure 2:**
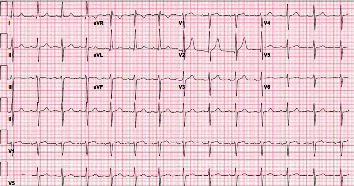
Patient's ECG three days before starting chemotherapy. ECG is normal with no ST segment or T wave abnormalities.

**Figure 3:**
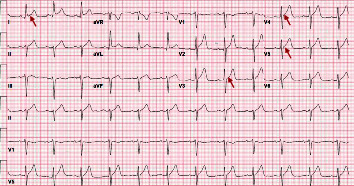
Patient's ECG after 2 hours from arrival to the ED. ECG shows new ST elevations on lead 1, V3, V4, and V5 (red arrows).

**Figure 4:**
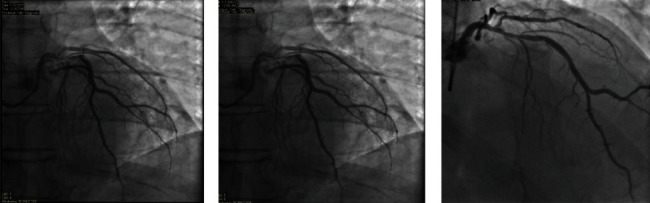
Pictures of emergent coronary angiogram after patient's arrival to the ED. Pictures show only mild focal left anterior descending artery disease.

**Figure 5:**
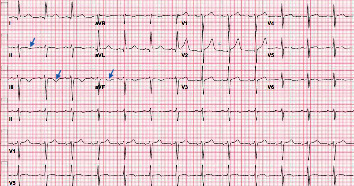
Patient's ECG after 6 hours of hospitalization. ECG shows that *T* waves are normalized in most of the leads, but it still remains inverted in lead II, III, aVF (blue arrows).

**Figure 6:**
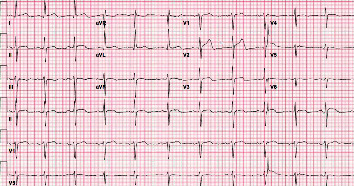
Patient's ECG after 24 hours of hospitalization. CG shows that *T* waves are normalized in most of the leads and there are no ST segment abnormalities.

**Figure 7:**
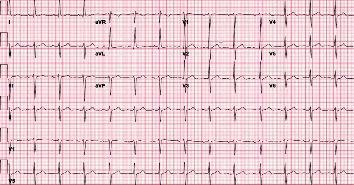
Patient's ECG on discharge ECG is normal with no *T* wave or ST segment abnormalities.

A 70-year-old male with newly diagnosed colorectal carcinoma presented to the emergency department with chest pain which developed during the first course of chemotherapy treatment with FOLFOX (a combination of oxaliplatin, leucovorin, and 5-FU). The patient informed his oncologist about the chest pain, who stopped his 5-FU infusion and sent him to the Emergency Department (ED). The pain was described as severe, squeezing in nature, located in the middle of the chest, without aggravating and alleviating factors. The patient did not have any known cardiac history or its risk factors like hypertension, hypercholesterolemia, or diabetes. Family history was negative for coronary artery disease, social history was not significant for any cardiac risk factors, and he was not a smoker and was not using any illicit drugs. Vitals were stable and physical examination was unremarkable. On initial ECG in the ED, the patient was found to have ischemic changes with diffuse *T* wave inversions and ST depressions in V3, V4, V5, and V6 (Figure 1), and the cardiac troponin I (cTnI) was elevated to 0.06 ng/ml (reference range 0–0.03 ng/ml). Of note, patient had ECG done during his regular visit to primary care physician three days before starting chemotherapy which was completely normal (Figure 2). Two hours later after arrival to the ED, ECG showed new ST elevations on lead 1, V3, V4, and V5 (Figure 3). Troponin increased from 0.06 ng/ml to 2.49 ng/ml and bedside echocardiogram demonstrated anterior wall hypokinesis. The patient was taken for an emergent coronary angiogram which revealed mild to moderate proximal left anterior descending artery disease with no acute occlusion (Figure 4). Diagnosis of 5-FU-induced coronary vasospasm was made. The patient was started on nitroglycerin and calcium channel blocker and admitted to a progressive care unit. Repeated serial ECGs showed gradual normalization of ST segment and ST and *T* wave abnormalities (Figures 5 and 6), troponin started to trend down and chest pain resolved in 24 hours. Two days later, the patient was discharged with normal ECG (Figure 7).

## 3. Discussion

The fluoropyrimidines, namely, 5-FU, is the third most commonly used chemotherapeutic agent for the treatment of solid tumors [6]. 5-FU is a pyrimidine analog that inhibits thymidylate synthase, an enzyme involved in DNA replication [7]. These agents function as S-phase antimetabolites, inducing double-strand DNA and single-strand DNA breaks and promote genomic instability; they interfere with DNA synthesis, repair, and elongation. 5-FU is the second most common agent after carboplatin which can cause cardiotoxicity [4]. Risk factors for 5-FU cardiotoxicity include older age, preceding history of cardiac disease, and concomitant use of cardiotoxic medications [8]. The most common clinical manifestation associated with cardiotoxicity is angina. Diagnosis of 5-FU cardiotoxicity mainly occurs during the first cycle of administration [9, 10], and it is mainly based on clinical presentation and symptoms, chest pain, elevated cardiac markers, electrocardiographic changes, changes of cardiac function by echocardiography, and results of coronary angiography. One prospective study [3] describes the cases of 5-FU-induced cardiotoxicity where the pain was the most common symptom, and serial cardiac enzyme levels were normal in all patients, also the symptoms immediately resolved after sensation of therapy and time to recovery was 5 to 60 minutes. In contrast, in our patient, we observed a significant elevation of troponin and the ECG changes resolved within 24 hours, which represents the different presentation of cardiac toxicity of 5-FU. The exact mechanism of cardiotoxicity is unknown; according to some theories, it can be due to coronary vasospasm, although myocardial infarction, arrhythmias, QT prolongation, heart failure, pericarditis, coronary dissection, and sudden cardiac death have also been reported [2, 5, 9]. In vitro models showed that 5-FU can cause concentration-dependent vasoconstriction of smooth muscle cells [11], but some studies have also shown that echocardiography demonstrated wall motion abnormalities in areas that do not correspond to the classic distribution of coronary arteries, that is, suggesting that a mechanism can be multifactorial [12]. For most patients with suspected fluoropyrimidine-induced chest pain, diagnostic coronary arteriography is indicated to exclude concomitant processes that account for acute coronary syndrome presentation and to guide treatment decisions. If coronary arteries are normal (or the extent of coronary artery disease is thought not to be clinically significant), a presumptive diagnosis of fluoropyrimidine cardiotoxicity can be made. Fluoropyrimidine treatment should be immediately discontinued if symptoms are suggestive of cardiotoxicity. Cardiac symptoms usually resolve, and cardiotoxicity appears to be completely reversible after cessation of therapy. Parenteral calcium channel blockers and long-acting nitrates can be used as a treatment if vasospasm is suspected. The presentation of cardiotoxicity in our patient also supports the vasospastic theory as the angiogram showed only mild clinically nonsignificant disease and the symptoms and ECG changes completely resolved after 24 hours of cessation of therapy and introduction of nitrates and calcium channel blockers. In general, the reintroduction of 5-FU after known cardiac toxicity is not recommended as the recurrence rate is as high as 90% and is associated with serious complications such as myocardial infarction, development of cardiogenic shock, and death [12, 13]; however, there are some exceptions when fluoropyrimidine challenge may be necessary for some patients in whom there are no alternative chemotherapy regiments or if the potential benefit is thought to outweigh the risk [14]. There is only limited literature available about rechallenging, and if this option is attempted, aggressive prophylaxis with aspirin, calcium channel blocker, and long-acting nitrates are required before administration of chemotherapy, along with informed consent, cardiology consultation, and close cardiac monitoring in an inpatient setting and immediate discontinuation if any sign of cardiotoxicity occurs. In 2015, FDA approved uridine triacetate for severe, life-threatening fluoropyrimidine toxicity; however, data is limited and there are only a few cases reports available when it was actually used [15, 16].

## 4. Conclusion

Our case emphasizes the importance of early recognition of the rare complication of the commonly used chemotherapeutic agent. In the majority of cases, 5-FU-induced coronary vasospasm is reversible; however, in view of the potentially lethal profile and successful clinical outcomes associated with early detection and intervention, physicians should be aware of its existence.
